# The Effect of Attending Steiner Schools during Childhood on Health in Adulthood: A Multicentre Cross-Sectional Study

**DOI:** 10.1371/journal.pone.0073135

**Published:** 2013-09-12

**Authors:** H. Felix Fischer, Sylvia Binting, Angelina Bockelbrink, Peter Heusser, Christoph Hueck, Thomas Keil, Stephanie Roll, Claudia Witt

**Affiliations:** 1 Institute for Social Medicine, Epidemiology and Health Economics, Charité University Medical Center, Berlin, Germany; 2 Chair for Theory of Medicine, Integrative and Anthroposophic Medicine, Faculty of Health, Witten/Herdecke University, Germany; 3 Freie Hochschule Stuttgart – Seminar für Waldorfpädagogik, Stuttgart, Germany; IUMSP, University Hospital Lausanne, Switzerland

## Abstract

**Background:**

It is speculated that attending Steiner schools, whose pedagogical principles include an account for healthy psycho-physical development, may have long-term beneficial health effects. We examined whether the current health status differed between former attendees of German Steiner schools and adults from the general population. Furthermore, we examined factors that might explain those differences.

**Methods:**

We included former Steiner school attendees from 4 schools in Berlin, Hanover, Nuremberg and Stuttgart and randomly selected population controls. Using a self-report questionnaire we assessed sociodemographics, current and childhood lifestyle and health status. Outcomes were self-reports on 16 diseases: atopic dermatitis, allergic rhinitis, bronchial asthma, chronic obstructive pulmonary disease (COPD), cardiac arrhythmia, cardiac insufficiency, angina pectoris, arteriosclerosis, hypertension, hypercholesterolemia, osteoarthritis, rheumatism, cancer, diabetes, depression and multiple sclerosis. Furthermore, participants rated the symptom burden resulting from back pain, cold symptoms, headache, insomnia, joint pain, gastrointestinal symptoms and imbalance. Unadjusted and adjusted odds ratios were calculated for each outcome.

**Results:**

1136 Steiner school attendees and 1746 controls were eligible for analysis. Both groups were comparable regarding sex, age and region, but differed in nationality and educational status. After adjusting for possible confounders, we found statistically significant effects of Steiner school attendance for osteoarthritis (OR 0.69 [0.49–0.97]) and allergic rhinitis (OR 0.77, [0.59–1.00]) as well as for symptom burden from back pain (OR 0.80, [0.64–1.00]), insomnia (OR 0.65, [0.50–0.84]), joint pain (OR 0.62, [0.48–0.82]), gastrointestinal symptoms (OR 0.76, [0.58–1.00]) and imbalance (OR 0.60, [0.38–0.93]).

**Conclusions:**

The risk of most examined diseases did not differ between former Steiner school attendees and the general population after adjustment for sociodemographics, current and childhood lifestyle features, but symptom burden from some current health complaints was reported less by former Steiner school attendees. Results must be interpreted with caution since the analysis was exploratory.

## Background

84,048 pupils and students in primary and secondary education (aged 5/6 to 19/20) attended 218 Steiner schools (‘Waldorf Schulen’) in 2010/11 in Germany and their number is growing [1]. Still, this is less than 1% of about 11.5 million German pupils and students in primary and secondary education [2]. The Steiner schools' curriculum is based on the anthroposophic background as developed by Rudolf Steiner [3], and it is claimed that these ideas and the corresponding anthroposophic lifestyle have positive effects on health [4]. According to the anthroposophic philosophy, the intellectual, emotional and volitional development of the child is associated with the physiological development of neurosensory, rhythmic (mainly cardio-respiratory) and metabolic-limb-functions [5]. Thus, a holistic pedagogical curriculum providing intellectual, aesthetic and motor activities in a balanced form [6] aims to promote a more healthy psycho-physiological constitution in general [4].

Results from a number of studies suggested that attending Steiner schools seemed to be a protective factor for the development of allergies [7]–[10]. It was hypothesized that this is an effect of anthroposophic lifestyle features such as restrictive use of antibiotics and others [9]–[13]. However, in the large Dutch KOALA birth cohort study no associations were found between early antibiotic intake and allergy development [14]–[16].

These studies mainly assessed the current health status of children in relation to an anthroposophic lifestyle [9], but in a survey of former Steiner School attendees, remarkable differences in prevalence of certain diseases were found in adulthood compared to the prevalence estimates derived from the German Health Survey [17]. However, methodological differences including study setting and mode of data collection make comparisons difficult. Uncertainties about the frequency of diseases and the current health status of former Steiner school attendees remain.

Our primary aim was to investigate whether the prevalence of common diseases and the current health status differ between former attendees of German Steiner schools and those of other German schools. Since the claim of Steiner Schools to promote a healthier psycho-physiological constitution is broad, we decided to evaluate a wide range of chronic diseases and symptoms. Our secondary aim was to identify sociodemographic, childhood and current lifestyle factors that might explain possible differences between both groups.

## Methods

### Study design, setting, and sample

The study was designed as a multicentre, cross-sectional survey comparing former Steiner School attendees with a random sample of the general population. The sample of Steiner school students was selected from registries of four Steiner schools in the North (Berlin, Hanover) and the South of Germany (Nuremberg, Stuttgart). These schools were selected because they are among the first Steiner schools established in Germany and therefore older school attendees could be included. We selected former Steiner school attendees aged 20 to 80 years per residential district based on postcodes beginning from the most frequent district until samples of at least 800 participants per school had been achieved. In order to protect privacy contact data was handled only at the schools, but for matching purposes, we assessed the number of included persons between the ages of 20–29, 30–49 and 50–80 years.

A random sample from the general population was drawn as a control group. Residents' Registration Offices provided contact data, matched to the distribution of postal codes and age groups of Steiner school graduates. A lower response in the general population compared to Steiner schools graduates was expected; therefore 60% more persons were contacted in the control group. Given the overall small number of Steiner schools, we did not expect a significant overlap between both samples.

The study was approved by the ethics committee of Charité University Medical Center, Berlin on July, 7th, 2010 under file number EA1/155/10.

### Assessment of sociodemographics and health behaviour

Study participants were asked to fill out a postal questionnaire, which was based on the German Health Survey [18], [19]. Reminders were sent by mail after five and ten weeks, respectively.

In addition to common sociodemographic information such as age, sex and nationality, the questionnaire included questions regarding childhood and parenting (educational status of parents, existence of siblings, parents in favour of certain pedagogic method, existence of spiritual or religious attitudes, amount of attention towards physical activity and balanced diet in childhood). We also assessed actual social support as operationalized in the German Health Survey with 4 items (number of close persons, interest of other people on oneself, availability of help from neighbours and possibility to make new acquaintances) [18], [19].

Health behaviour was recorded by self reports on alcohol consumption, smoking, eating fresh fruits and vegetables, physical activity, and ratings of attention towards physical activity and balanced diet on Likert scales with 4 to 6 levels as in the German Health Survey. For the analyses, these ratings were dichotomized at their respective middle category as discussed among authors.

### Assessment of health status

Outcomes of the study were self-reports on 16 diseases diagnosed at least once by a physician: atopic dermatitis, allergic rhinitis, bronchial asthma, chronic obstructive pulmonary disease (COPD), cardiac arrhythmia, cardiac insufficiency, angina pectoris, arteriosclerosis, hypertension, hypercholesterolemia, osteoarthritis, rheumatism, cancer, diabetes, depression, and multiple sclerosis. Furthermore, participants were asked to rate the burden resulting from 7 symptoms: back pain, cold symptoms, headache, insomnia, joint pain, gastrointestinal symptoms, and imbalance on a 5 point Likert scale (not at all, a little, moderate, rather strong, very strong). For analysis these ratings were dichotomized into “less than moderate” and “moderate and above”. Body mass index (BMI) was calculated from height and weight as reported by participants. Participants were asked about the extent they care about their health, the extent of use of certain forms of complementary and alternative medicine (CAM) now and in their childhood, and the number of days spent in hospital within the last year.

### Statistical Analysis

Sample size calculation was conducted using the prevalence of hypertension (28.5%), osteoarthritis (19.0%) and diabetes (7.0%) as described in the German Health Survey for the general population [20]. We aimed to include 2000 participants per group in order to detect differences between the Steiner schools attendees and the general population of 4%, 3.5%, and 2% in prevalence of hypertension, osteoarthritis and diabetes respectively with 80% power and a two-sided significance level of 5%. We did not adjust results for multiple comparisons, thus all results are considered exploratory.

Categorical sociodemographic variables were tested for equal distribution between both groups by a chi-square-test, continuous variables for equal means by t-tests. We present prevalence estimates with 95% confidence interval (CI, in brackets) for the diseases and symptoms. For the outcomes (prevalence of disease or burden of symptoms > =  moderate), we used an unadjusted logistic regression model for each outcome to compare the two groups (Model 1). Analysis was also adjusted for age, sex and region (Model 2) and additionally for variables possibly responsible for the difference between both groups (Model 3). It was predefined that one model should be developed for all diseases and symptoms to allow comparison of the effect of attending a Steiner school over all outcomes. Those variables should be included that 1) showed relevant group differences in the descriptive data, and 2) that were possible influencing factors for at least one of the diseases or symptoms. The variables were determined in a two-step consensus procedure. Firstly, two medical doctors/epidemiologists, one statistician, one psychologist and one data manager developed the model. In a second step this model was discussed with and approved by the advisory board.

We present unadjusted and adjusted odds ratios (and 95% CI) for the odds of an outcome in the Steiner school group with respect to the control group. For the fully adjusted Model 3 we also report the association of the adjustment variables and the outcomes to identify factors that might explain possible group differences.

We performed three sensitivity analyses in relation to the fully adjusted Model 3, where we (1) additionally included BMI as a possible confounder relevant to the development of many chronic diseases, (2) included only German citizens and (3) included only persons in the Steiner school group which reported to have attended a Steiner school full time between the ages of 7 and 14 (which is considered by anthroposophic theory as an important time span in the development of health).

A statistical analysis plan (SAP) was written by H.F.F., T.K., S.R., and C.W., and agreed by the advisory board before the start of the data analyses. All statistical analyses were conducted in R 2.15 [21], based on the full data set with cases with missing values excluded from each analysis separately. As a post-hoc sensitivity analysis we imputed and analysed 100 complete datasets using the R-Package mice [22].

## Results

A final sample of 2882 persons was eligible for the analysis. Overall, 8614 questionnaires were sent out by mail, 3291 to former Steiner schools attendees and 5269 to the control group of the general population. We received 2933 questionnaires (1147 Steiner group and 1786 control group) and had to exclude 12 persons, who were outside the predefined age range (11 Steiner, 1 control). Furthermore, 39 persons from the control group, who reported having attended a Steiner school, were excluded from the analyses.

Overall, the response was similar in both groups (34.8% Steiner group, 33.5% control group). About 90% of the non-responders reported no reason for denial of participation; in most of the remaining cases current addresses had changed and questionnaires were sent back. In the control group, response rate was lower in males compared to females (29.5% vs. 37.5%, p<0.001) and increased with age (age group 20–29 years 27.1%; 30–49 32.5%, 50–80 42.6%, p<0.001). Information on age and sex of Steiner non-responders was not available, because data of the Steiner school group were handled in the respective schools.

The variables with the highest number of missing values in the questionnaire were “focus on balanced diet in childhood” and “focus on physical activity in childhood” with 96 (3.3%) respectively 126 (4.4%) missing values. In general, controls were found to have slightly more missing responses than Steiner school attendees (0.16 vs. 0.11 missing values per participant, difference  = 0.05 [−0.01–0.10], p = 0.07).

While distribution of age, sex and family status was similar in both groups, former Steiner school attendees as well as their parents had a higher educational status compared to controls. Almost all (99.2%) former Steiner school attendees held German citizenship compared to 93.0% in the control group. Steiner school attendees reported more often focus on balanced diet and physical activities by their parents in their childhood than the control subjects did (Table 1).

**Table 1:** Baseline characteristics.

	Characteristic	N (valid cases)	Former Steiner school attendees n(%)^1^	Controls n(%)^1^
*Sociodemographics*				
	Age Mean (sd)	2882	44.3 (16.4)	44.9 (17.0)
	Sex	2876		
	Female		669 (59.2%)	994 (56.9%)
	Male		461 (40.8%)	752 (43.1%)
	Region	2882		
	Berlin		261 (23.0%)	430 (24.6%)
	Hanover		252 (22.2%)	469 (26.9%)
	Nuremberg		284 (25.0%)	407 (23.3%)
	Stuttgart		339 (29.8%)	440 (25.2%)
	Nationality	2877		
	German		1084 (95.7%)	1583 (90.8%)
	German and other		40 (3.5%)	39 (2.2%)
	not German		9 (0.8%)	122 (7.0%)
	Education^2^	2875		
	A-level not achieved		265 (23.4%)	758 (43.5%)
	A-level achieved		869 (76.6%)	983 (56.5%)
	Family Status:	2868		
	In relationship		794 (70.3%)	1204 (69.2%)
	Single		335 (29.7%)	535 (30.8%)
	Social support^3^ Mean (sd)	2860	11.3 (2.3)	10.5 (2.5)
*Actual lifestyle features*				
	Alcohol consumption:	2861		
	less than moderate		741 (65.6%)	1163 (67.1%)
	moderate and more		388 (34.4%)	569 (32.8%)
	Smoking	2882		
	current smoker		280 (24.6%)	471 (27.0%)
	former or never smoker		856 (75.3%)	1275 (73.0%)
	Attention on balanced diet	2856		
	less than moderate		113 (10.0%)	328 (19.0%)
	moderate and more		1015 (90.0%)	1400 (81.0%)
	Intake of fresh vegetables and fruits on	2863		
	on less than 5 days/week		321 (28.4%)	714 (41.2%)
	on 5 days/week or more		808 (71.6%)	1020 (58.8%)
	Attention on physical activity	2861		
	less than moderate		606 (53.7%)	884 (51.0%)
	moderate and more		523 (46.3%)	848 (49.0%)
	Physical activity	2860		
	on less than 5 days/week		885 (78.5%)	1455 (84.0%)
	on 5 days/week or more		243 (21.5%)	277 (16.0%)
*Childhood lifestyle features*				
	Education Parents^2^	2882		
	A-Level or university degree		857 (75.4%)	736 (42.1%)
	below A-Level		260 (22.9%)	905 (51.8%)
	Others/unknown		19 (1.7%)	105 (6.0%)
	Siblings	2863		
	none		162 (14.3%)	263 (15.2%)
	at least one		969 (85.7%)	1469 (84.8%)
	Parents favoured certain pedagogic method	2882		
	yes		1004 (88.4%)	407 (23.3%)
	no		108 (9.5%)	1209 (69.2%)
	unknown		24 (2.1%)	130 (7.4%)
	Parents had spiritual or religious beliefs	2869		
	yes		660 (58.4%)	722 (41.5%)
	no		439 (38.8%)	992 (57.0%)
	unknown		31 (2.7%)	25 (1.4%)
	Focus on balanced diet in childhood	2786		
	less than moderate		181 (16.3%)	578 (34.5%)
	moderate and more		931 (83.7%)	1096 (65.5%)
	Focus on physical activity in childhood less than moderate	2756		
	less than moderate		228 (20.7%)	439 (26.5%)
	moderate and more		872 (79.3%)	1217 (73.5%)

1 except Age and Social support (mean (sd)).

2 A-level is equivalent to German (Fach-)Abitur as necessary for university attendance.

3 0–16 with higher values meaning more social support.

**Figure 1:**
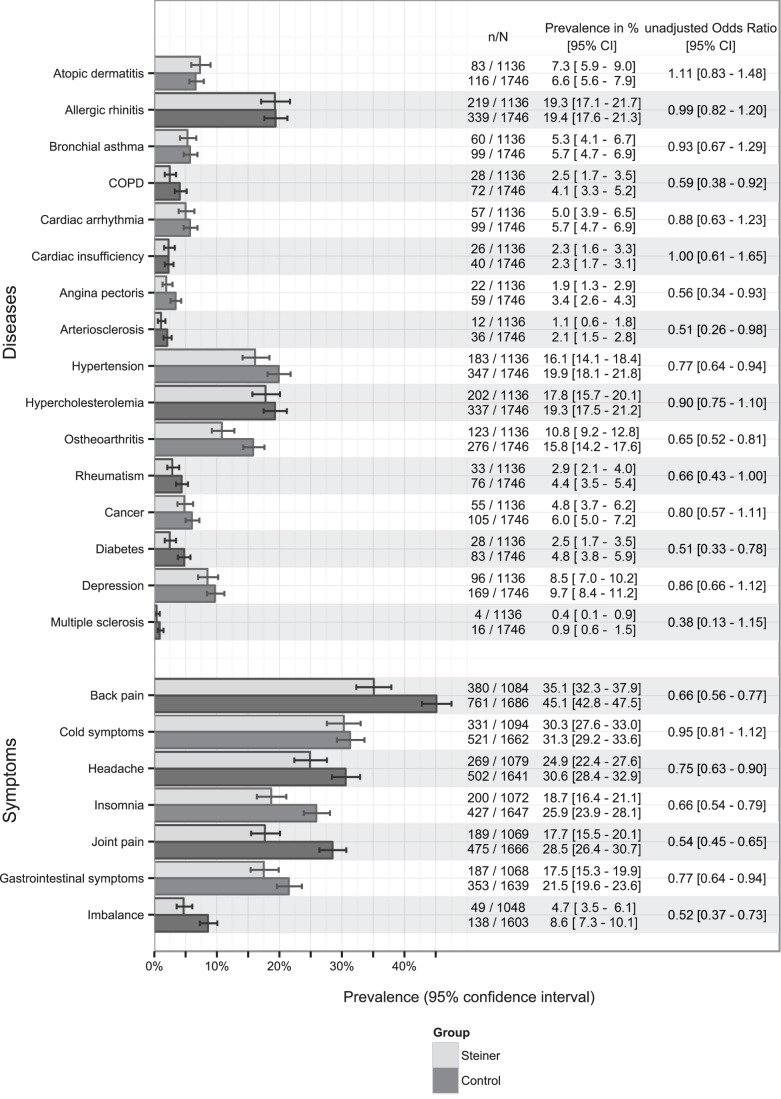
Disease and symptom prevalence estimates including 95% confidence intervals and unadjusted odds ratios.

Most prevalent diseases were hypertension (16.1% [14.1%–18.4%]) in Steiner group compared to 19.9% [18.1%–21.8%] in control group, allergic rhinitis (19.3% [17.1%–21.7%] versus 19.4% [ 17.6%–21.3%]), hypercholesterinaemia (17.8% [15.7%–20.1%] versus 19.3% [17.5%–21.2%]) and osteoarthritis (10.8% [9.2%–12.8%] versus 15.8% [14.2%–17.6%]). Back pain, headache and joint pain were most often reported to cause moderate or worse symptom burden. In general, for several diseases and symptoms, reported prevalence was lower in former Steiner school attendees compared to the general population. Unadjusted odds ratios of disease prevalence ranged from 0.38 [0.13–1.15] for multiple sclerosis to 1.11 [0.83–1.48] for atopic dermatitis and for symptom burden from 0.52 [0.37–0.73] for imbalance to 0.95 [0.81–1.12] for cold symptoms (Figure 1).

There was no significant difference between the Steiner school and the control group in their rating about the extent to which they care about health (p = 0.592), but former Steiner school attendees had lower mean BMI (23.5 vs. 24.6, difference 1.1 [0.75–1.39], p<0.001) and had spent less days in hospitals within the last 12 months (0.79 vs. 1.63, difference 0.84 [0.30–1.38] days, p<0.001). Also, while 54% (598 of 1113) of the Steiner school group use CAM moderate or more often, this was found for 25% (420 out of 1703) of the controls (p<0.001).

**Figure 2:**
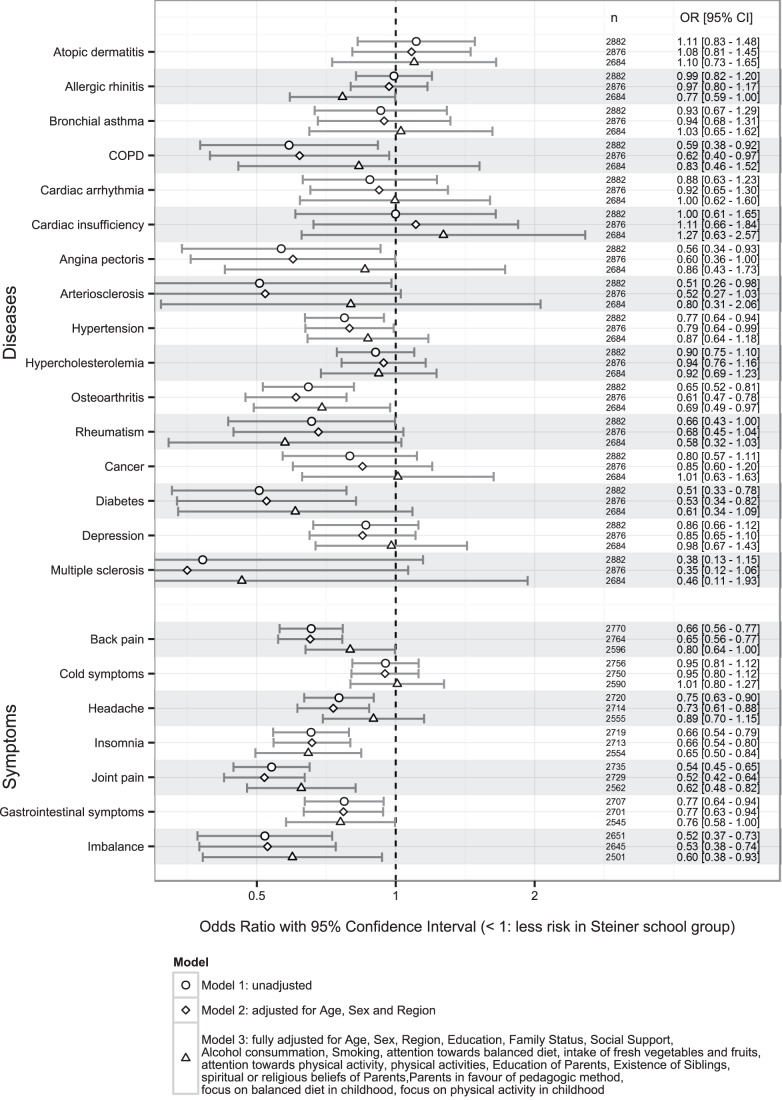
Comparison of unadjusted and adjusted effect estimates (odds ratios) on outcome variables.

Based on discussion within the scientific study committee about group differences and their possible impact on outcomes, we decided after an initial adjustment for age, sex, and region (Model 2) to adjust additionally for variables of parents and childhood (parental educational status, siblings, spiritual/religious beliefs, in favour of pedagogic method, CAM use, focus on balanced diet and physical activity), personal educational status, social support (family status and social support) and health behaviour (alcohol consumption, smoking, intake of fresh fruits and vegetables, focus on balanced diet and physical activity) (Model 3). These adjusted odds ratios for Steiner school attendance for disease prevalence range from 0.46 [0.11–1.93] for multiple sclerosis to 1.27 [0.63–2.57] for cardiac insufficiency and for reported symptom burden from 0.60 [0.38–0.93] for imbalance to 1.01 [0.80–1.27] for common cold symptoms (Figure 2). The difference of BMI was 0.39 [−0.01–0.80] in favour of Steiner schools (p = 0.06) in adjusted analysis (Model 2).

All odds ratios for each variable in Model 3 are reported in Table 2 and Table 3. For example, in the adjusted analysis the odds ratio for former Steiner school attendees compared to controls for hypertension is 0.87; its confidence interval ranges between 0.64 and 1.18. Age (OR 1.08 [1.07–1.09]) and sex (male compared to female) (OR 1.48 [1.16–1.89]) were associated with a higher risk of hypertension, whereas answering the item “How much attention do you draw towards physical activity” with “moderately and more” was associated with lower risk of hypertension compared to those who gave less attention to physical activity (OR 0.71 [0.56–0.91]). Similarly, answering “How much attention do you draw towards balanced diet” with “moderately and more” was also associated with a lower risk of hypertension (OR 0.72 [0.52–1.01]). Self-reports on actual behaviour like physical activities on more than 5 days a week (OR 1.04 [ 0.77–1.42]) and intake of fresh vegetables on more than 5 days a week (OR 0.96 [0.74–1.24]) was not associated with hypertension.

**Table 2:** Odds ratios of Steiner school attendance, sociodemographic variables, current and childhood lifestyle factors on diseases; from multivariable logistic regression including all factors listed (fully adjusted model 3).

			Sociodemographics							Current lifestyle variables							Childhood lifestyle variables								
	Valid cases in model	Steiner School	Age (per year)	Sex: Male	Region: Hanover	Region: Nuremberg	Region: Stuttgart	Education: A-level	Family Status: Single	Social Support	Alcohol consumption: moderate 5.5and more	Smoker	Attention balanced diet: moderate and more	Fresh vegetables and fruits: 5–7 days per week	Attention physical activity: moderate and more	Physical activities: 5–7 days per week	No siblings	Education Parents: below A-Level	Education Parents: Others/unknown	Parents favour pedagogic method: no	Parents favour pedagogic method: unknown	Parents spiritual or religious beliefs no	Parents spiritual or religious beliefs: unknown	Focus on balanced diet in childhood: moderate and more	Focus on physical activity in childhood: moderate and more
Atopic dermatitis	2684	1.10 (0.73–1.65)	0.98 (0.97–0.99)	0.56 (0.40–0.78)	0.86 (0.56–1.32)	0.83 (0.54–1.25)	0.96 (0.64–1.44)	1.00 (0.69–1.43)	1.09 (0.79–1.50)	0.98 (0.92–1.05)	0.84 (0.61–1.16)	1.11 (0.79–1.56)	1.51 (0.91–2.49)	0.97 (0.69–1.37)	1.02 (0.75–1.40)	0.95 (0.64–1.41)	1.09 (0.73–1.64)	0.81 (0.57–1.16)	0.85 (0.35–2.09)	1.10 (0.72–1.67)	1.06 (0.47–2.36)	0.89 (0.65–1.22)	1.20 (0.45–3.15)	0.80 (0.54–1.19)	1.17 (0.80–1.71)
Allergic rhinitis	2684	0.77 (0.59–1.00)	0.99 (0.98–0.99)	1.22 (0.99–1.50)	1.09 (0.81–1.45)	1.18 (0.89–1.57)	1.28 (0.97–1.68)	1.18 (0.93–1.50)	0.85 (0.68–1.05)	1.04 (1.00–1.09)	0.83 (0.67–1.02)	0.66 (0.52–0.84)	1.41 (1.03–1.93)	1.07 (0.86–1.34)	0.86 (0.70–1.06)	0.95 (0.73–1.23)	1.27 (0.97–1.65)	1.04 (0.83–1.31)	0.78 (0.42–1.47)	0.83 (0.64–1.08)	0.69 (0.39–1.21)	0.89 (0.73–1.09)	0.75 (0.34–1.62)	0.75 (0.59–0.96)	1.03 (0.81–1.30)
Bronchial asthma	2684	1.03 (0.65–1.62)	1.00 (0.98–1.01)	1.31 (0.92–1.87)	1.04 (0.64–1.68)	0.93 (0.57–1.51)	0.89 (0.55–1.44)	1.04 (0.69–1.55)	0.84 (0.57–1.24)	0.98 (0.91–1.05)	0.94 (0.65–1.35)	0.89 (0.59–1.33)	1.10 (0.65–1.86)	1.03 (0.71–1.51)	0.99 (0.69–1.42)	0.99 (0.63–1.55)	0.99 (0.62–1.59)	1.11 (0.75–1.65)	0.78 (0.27–2.29)	0.97 (0.61–1.55)	1.15 (0.49–2.70)	1.16 (0.81–1.66)	0.72 (0.17–3.07)	1.14 (0.73–1.77)	0.92 (0.61–1.39)
COPD	2684	0.83 (0.46–1.52)	1.04 (1.02–1.06)	0.68 (0.42–1.09)	1.19 (0.67–2.14)	0.46 (0.22–0.97)	0.92 (0.49–1.70)	0.87 (0.53–1.42)	1.12 (0.70–1.77)	0.95 (0.87–1.03)	1.17 (0.72–1.91)	1.78 (1.11–2.86)	0.74 (0.42–1.32)	0.56 (0.35–0.90)	0.60 (0.37–0.97)	1.23 (0.69–2.19)	0.88 (0.48–1.60)	1.18 (0.71–1.97)	1.15 (0.44–2.99)	1.05 (0.58–1.89)	2.06 (0.87–4.84)	1.08 (0.68–1.69)	1.04 (0.23–4.68)	1.02 (0.61–1.72)	0.89 (0.55–1.44)
Cardiac arrhythmia	2684	1.00 (0.62–1.60)	1.05 (1.03–1.06)	1.26 (0.85–1.85)	0.85 (0.49–1.46)	0.82 (0.46–1.45)	1.03 (0.61–1.73)	0.97 (0.64–1.48)	1.20 (0.81–1.78)	0.87 (0.81–0.93)	1.19 (0.79–1.79)	0.67 (0.41–1.10)	1.25 (0.72–2.15)	0.97 (0.64–1.46)	0.89 (0.61–1.31)	0.93 (0.56–1.55)	1.52 (0.97–2.37)	1.05 (0.69–1.61)	1.08 (0.44–2.67)	0.91 (0.56–1.47)	1.06 (0.45–2.50)	0.89 (0.61–1.30)	0.34 (0.04–2.55)	0.80 (0.52–1.22)	1.11 (0.73–1.69)
Cardiac insufficiency	2684	1.27 (0.63–2.57)	1.06 (1.04–1.08)	1.20 (0.67–2.15)	0.51 (0.25–1.04)	0.55 (0.26–1.18)	0.30 (0.13–0.70)	0.63 (0.33–1.19)	1.41 (0.79–2.51)	0.96 (0.86–1.06)	1.58 (0.81–3.09)	0.34 (0.13–0.88)	0.74 (0.35–1.56)	0.74 (0.41–1.35)	0.64 (0.35–1.15)	0.55 (0.21–1.43)	1.22 (0.60–2.45)	0.67 (0.34–1.30)	1.36 (0.44–4.18)	1.07 (0.52–2.20)	0.82 (0.22–3.14)	1.20 (0.68–2.12)	0.00 (0.00–Inf)	0.98 (0.51–1.89)	1.40 (0.74–2.67)
Angina pectoris	2684	0.86 (0.43–1.73)	1.08 (1.06–1.11)	2.34 (1.33–4.10)	0.77 (0.36–1.63)	0.90 (0.41–1.97)	0.57 (0.25–1.28)	0.53 (0.29–0.99)	0.93 (0.51–1.70)	0.98 (0.88–1.08)	0.86 (0.48–1.52)	1.40 (0.74–2.65)	0.74 (0.35–1.54)	1.37 (0.76–2.46)	0.46 (0.26–0.82)	1.84 (0.94–3.60)	1.12 (0.57–2.21)	1.20 (0.63–2.27)	1.73 (0.59–5.10)	1.40 (0.69–2.83)	1.77 (0.61–5.16)	0.91 (0.53–1.55)	0.00 (0.00-Inf)	1.21 (0.65–2.25)	1.09 (0.60–1.98)
Arteriosclerosis	2684	0.80 (0.31–2.06)	1.11 (1.07–1.15)	1.93 (0.91–4.07)	1.77 (0.46–6.92)	5.45 (1.42–20.90)	1.77 (0.43–7.27)	1.14 (0.47–2.74)	0.66 (0.28–1.57)	0.95 (0.83–1.08)	4.13 (1.51–11.28)	1.82 (0.76–4.37)	1.09 (0.39–3.06)	0.86 (0.40–1.81)	0.43 (0.20–0.92)	1.56 (0.60–4.05)	0.84 (0.30–2.33)	1.00 (0.41–2.46)	2.86 (0.71–11.53)	1.89 (0.74–4.80)	0.72 (0.13–3.98)	0.66 (0.31–1.37)	0.00 (0.00-Inf)	0.90 (0.40–2.02)	1.04 (0.48–2.29)
Hypertension	2684	0.87 (0.64–1.18)	1.08 (1.07–1.09)	1.48 (1.16–1.89)	1.13 (0.80–1.57)	0.94 (0.66–1.35)	0.86 (0.61–1.20)	0.93 (0.72–1.20)	1.14 (0.89–1.48)	0.97 (0.92–1.01)	0.86 (0.68–1.11)	0.93 (0.70–1.23)	0.72 (0.52–1.01)	0.96 (0.74–1.24)	0.71 (0.56–0.91)	1.04 (0.77–1.42)	0.99 (0.72–1.35)	1.20 (0.92–1.55)	0.82 (0.45–1.50)	1.00 (0.74–1.36)	0.87 (0.49–1.56)	1.04 (0.82–1.32)	1.20 (0.52–2.74)	1.14 (0.87–1.50)	0.83 (0.64–1.08)
Hypercholesterolemia	2684	0.92 (0.69–1.23)	1.06 (1.05–1.07)	1.28 (1.01–1.61)	0.91 (0.66–1.26)	1.07 (0.76–1.49)	0.99 (0.72–1.35)	0.89 (0.69–1.14)	1.23 (0.97–1.57)	0.99 (0.95–1.04)	1.08 (0.85–1.37)	1.00 (0.77–1.31)	0.93 (0.67–1.28)	1.04 (0.81–1.33)	0.63 (0.50–0.80)	0.94 (0.70–1.28)	0.89 (0.66–1.21)	0.99 (0.77–1.27)	0.97 (0.55–1.68)	0.95 (0.71–1.27)	1.06 (0.62–1.83)	0.83 (0.66–1.05)	0.77 (0.33–1.76)	0.84 (0.65–1.09)	1.01 (0.79–1.31)
Osteoarthritis	2684	0.69 (0.49–0.97)	1.08 (1.07–1.10)	0.68 (0.51–0.90)	0.88 (0.58–1.33)	1.57 (1.04–2.37)	1.43 (0.97–2.12)	0.92 (0.69–1.24)	0.99 (0.74–1.32)	0.96 (0.91–1.01)	0.98 (0.74–1.31)	1.11 (0.81–1.53)	0.78 (0.53–1.15)	1.07 (0.79–1.44)	0.91 (0.70–1.20)	1.37 (0.98–1.91)	0.93 (0.65–1.33)	1.26 (0.94–1.69)	1.09 (0.58–2.07)	1.10 (0.78–1.54)	1.07 (0.57–1.98)	1.00 (0.76–1.31)	1.17 (0.47–2.95)	0.91 (0.67–1.24)	1.52 (1.12–2.07)
Rheumatism	2684	0.58 (0.32–1.03)	1.06 (1.05–1.08)	0.70 (0.44–1.13)	1.01 (0.52–1.98)	0.91 (0.44–1.89)	1.62 (0.86–3.03)	1.03 (0.62–1.70)	1.71 (1.10–2.66)	0.96 (0.88–1.04)	1.09 (0.66–1.80)	1.34 (0.81–2.24)	0.53 (0.29–0.96)	0.98 (0.60–1.61)	0.61 (0.39–0.98)	1.69 (0.99–2.87)	0.78 (0.41–1.48)	0.90 (0.54–1.50)	1.66 (0.70–3.91)	0.72 (0.41–1.27)	1.47 (0.63–3.46)	0.97 (0.62–1.53)	1.64 (0.46–5.81)	1.30 (0.76–2.21)	0.87 (0.53–1.41)
Cancer	2684	1.01 (0.63–1.63)	1.07 (1.05–1.08)	0.86 (0.58–1.28)	1.56 (0.91–2.67)	0.81 (0.43–1.52)	1.04 (0.59–1.84)	0.80 (0.53–1.22)	1.15 (0.78–1.71)	1.03 (0.96–1.11)	1.41 (0.93–2.14)	1.17 (0.74–1.84)	0.83 (0.48–1.43)	0.77 (0.51–1.15)	1.06 (0.72–1.55)	0.79 (0.47–1.34)	0.96 (0.58–1.60)	0.93 (0.60–1.42)	2.26 (1.07–4.76)	1.18 (0.73–1.90)	0.41 (0.13–1.26)	0.99 (0.68–1.44)	0.98 (0.22–4.37)	1.22 (0.78–1.91)	1.01 (0.66–1.54)
Diabetes	2684	0.61 (0.34–1.09)	1.08 (1.06–1.10)	1.62 (1.01–2.60)	0.88 (0.46–1.67)	0.69 (0.33–1.41)	1.09 (0.58–2.07)	0.71 (0.43–1.17)	1.25 (0.78–2.00)	1.02 (0.93–1.11)	1.24 (0.77–2.01)	1.46 (0.87–2.44)	0.63 (0.35–1.14)	0.68 (0.42–1.09)	0.77 (0.49–1.22)	0.98 (0.53–1.85)	1.53 (0.90–2.60)	0.91 (0.55–1.52)	0.95 (0.35–2.56)	0.89 (0.51–1.57)	1.00 (0.38–2.58)	1.33 (0.84–2.10)	2.97 (0.91–9.73)	1.12 (0.66–1.89)	1.03 (0.62–1.69)
Depression	2684	0.98 (0.67–1.43)	1.01 (1.00–1.02)	0.43 (0.32–0.59)	0.70 (0.46–1.06)	0.92 (0.62–1.35)	0.91 (0.62–1.33)	1.20 (0.86–1.66)	1.34 (1.00–1.79)	0.83 (0.79–0.88)	0.83 (0.62–1.12)	1.92 (1.42–2.60)	1.19 (0.80–1.78)	0.72 (0.53–0.98)	0.80 (0.60–1.08)	1.19 (0.82–1.72)	1.18 (0.81–1.70)	0.95 (0.69–1.32)	1.55 (0.82–2.96)	0.97 (0.66–1.40)	0.58 (0.26–1.29)	0.73 (0.55–0.98)	0.62 (0.21–1.81)	0.74 (0.53–1.04)	0.81 (0.59–1.10)
Multiple sclerosis	2684	0.46 (0.11–1.93)	1.00 (0.96–1.03)	0.64 (0.22–1.84)	0.54 (0.10–3.10)	0.98 (0.23–4.15)	2.30 (0.66–8.07)	0.61 (0.21–1.80)	1.26 (0.47–3.36)	0.93 (0.77–1.11)	1.88 (0.57–6.13)	1.64 (0.60–4.53)	0.59 (0.18–2.00)	0.75 (0.26–2.14)	0.36 (0.11–1.17)	0.38 (0.05–2.96)	0.58 (0.13–2.61)	0.92 (0.31–2.77)	0.75 (0.08–7.17)	0.89 (0.26–3.01)	0.68 (0.07–6.88)	1.56 (0.58–4.22)	0.00 (0.00-Inf)	1.48 (0.44–4.98)	1.51 (0.47–4.84)

Age and Social support (higher values mean higher social support) are continuous variables.

Comparison category for categorical variables: Sex: Female, Region: Berlin, Education: below A-levels, Family status: in relationship, alcohol consumption less than moderate, non-smoker, attention to balanced diet less than moderate, intake of fresh vegetables and fruits on less than 5 days a week, attention physical activity less than moderate, Education of parents: A-levels, attention to balanced diet in childhood less than moderate, attention to physical activity in childhood less than moderate, Parents in favour of pedagogic method: yes, spiritual or religious beliefs of parents: yes.

**Table 3:** Odds ratios of Steiner school attendance, sociodemographic variables, current and childhood lifestyle factors on diseases; from multivariable logistic regression including all factors listed (fully adjusted model 3).

			Sociodemographics							Actual lifestyle variables							Childhood lifestyle variables								
	Valid cases in model	Steiner School	Age (per year)	Sex: Male	Region: Hanover	Region: Nuremberg	Region: Stuttgart	Education: A-level	Family Status: Single	Social Support	Alcohol consummation: moderate and more	Smoker	Attention balanced diet: moderate and more	Fresh vegetables and fruits: 5–7 days per week	Attention physical activity: moderate and more	Physical activities: 5–7 days per week	No siblings	Education Parents: below A-Level	Education Parents: Others/unknown	Parents favour pedagogic method: no	Parents favour pedagogic method: unknown	Parents spiritual or religious beliefs no	Parents spiritual or religious beliefs: unknown	Focus on balanced diet in childhood: moderate and more	Focus on physical activity in childhood: moderate and more
Back pain	2596	0.80 (0.64–1.00)	1.01 (1.00–1.02)	0.62 (0.52–0.74)	1.17 (0.92–1.48)	1.32 (1.04–1.67)	1.06 (0.84–1.34)	0.78 (0.64–0.94)	0.94 (0.78–1.13)	0.94 (0.90–0.97)	0.94 (0.79–1.13)	1.40 (1.15–1.69)	0.96 (0.75–1.23)	0.83 (0.69–0.99)	0.72 (0.61–0.86)	1.08 (0.86–1.34)	0.98 (0.78–1.23)	1.15 (0.95–1.39)	1.26 (0.80–1.97)	1.00 (0.80–1.25)	1.34 (0.88–2.04)	1.01 (0.85–1.20)	1.18 (0.66–2.12)	0.90 (0.73–1.11)	1.00 (0.82–1.21)
Cold symptoms	2590	1.01 (0.80–1.27)	0.99 (0.98–0.99)	0.87 (0.73–1.05)	0.95 (0.74–1.21)	0.86 (0.68–1.10)	0.75 (0.59–0.95)	1.23 (1.00–1.51)	1.09 (0.90–1.32)	0.95 (0.92–0.99)	0.91 (0.76–1.10)	1.33 (1.09–1.62)	1.15 (0.88–1.49)	1.07 (0.88–1.30)	0.88 (0.73–1.06)	0.86 (0.68–1.08)	1.01 (0.79–1.28)	1.11 (0.91–1.35)	2.11 (1.33–3.33)	0.96 (0.76–1.21)	1.33 (0.86–2.06)	0.99 (0.83–1.19)	1.36 (0.74–2.49)	0.87 (0.70–1.08)	1.06 (0.86–1.31)
Headache	2555	0.89 (0.70–1.15)	0.97 (0.97–0.98)	0.41 (0.33–0.50)	1.24 (0.95–1.61)	0.88 (0.68–1.15)	0.97 (0.75–1.26)	0.80 (0.65–1.00)	0.92 (0.75–1.12)	0.93 (0.90–0.97)	1.09 (0.89–1.34)	1.12 (0.91–1.39)	0.90 (0.69–1.18)	0.76 (0.62–0.93)	0.73 (0.60–0.88)	0.90 (0.70–1.16)	1.14 (0.89–1.46)	1.14 (0.92–1.42)	1.38 (0.85–2.25)	0.91 (0.71–1.17)	0.90 (0.56–1.43)	0.97 (0.80–1.18)	1.33 (0.71–2.50)	0.92 (0.72–1.16)	0.94 (0.75–1.17)
Insomnia	2554	0.65 (0.50–0.84)	1.02 (1.01–1.02)	0.63 (0.51–0.78)	1.14 (0.86–1.50)	0.99 (0.75–1.32)	0.98 (0.74–1.30)	0.85 (0.68–1.06)	1.23 (1.00–1.52)	0.89 (0.86–0.93)	0.99 (0.80–1.23)	1.19 (0.95–1.50)	0.92 (0.69–1.23)	0.85 (0.68–1.05)	0.70 (0.56–0.86)	1.20 (0.92–1.55)	1.14 (0.88–1.48)	1.15 (0.92–1.44)	1.43 (0.88–2.32)	0.75 (0.58–0.97)	0.95 (0.59–1.52)	0.95 (0.77–1.16)	1.19 (0.60–2.36)	1.12 (0.88–1.44)	0.95 (0.75–1.19)
Joint pain	2562	0.62 (0.48–0.82)	1.05 (1.04–1.05)	0.93 (0.75–1.14)	1.07 (0.80–1.45)	1.24 (0.92–1.67)	1.04 (0.77–1.39)	0.67 (0.54–0.84)	1.03 (0.82–1.29)	0.96 (0.92–1.00)	1.11 (0.89–1.39)	1.34 (1.06–1.70)	0.91 (0.68–1.23)	0.85 (0.68–1.06)	0.71 (0.57–0.88)	1.25 (0.96–1.63)	0.91 (0.69–1.20)	1.00 (0.79–1.25)	1.36 (0.84–2.21)	1.01 (0.77–1.31)	1.24 (0.77–2.01)	1.09 (0.88–1.35)	1.95 (1.00–3.81)	1.04 (0.81–1.32)	1.02 (0.80–1.29)
Gastrointestinal symptoms	2545	0.76 (0.58–1.00)	1.00 (0.99–1.01)	0.72 (0.58–0.90)	1.00 (0.75–1.34)	1.09 (0.82–1.45)	0.96 (0.72–1.27)	0.92 (0.73–1.17)	1.15 (0.92–1.43)	0.92 (0.88–0.96)	1.03 (0.83–1.28)	1.17 (0.93–1.47)	1.03 (0.77–1.38)	0.86 (0.69–1.08)	0.67 (0.54–0.83)	0.83 (0.62–1.10)	0.92 (0.70–1.22)	0.83 (0.66–1.05)	1.31 (0.80–2.14)	0.85 (0.65–1.11)	1.07 (0.66–1.74)	0.95 (0.77–1.17)	0.97 (0.47–2.02)	0.93 (0.72–1.19)	1.09 (0.85–1.38)
Imbalance	2501	0.60 (0.38–0.93)	1.03 (1.02–1.04)	0.71 (0.50–1.01)	0.95 (0.60–1.50)	0.89 (0.55–1.42)	0.69 (0.43–1.11)	0.87 (0.60–1.26)	1.30 (0.92–1.83)	0.92 (0.87–0.98)	1.45 (0.99–2.12)	1.02 (0.69–1.50)	1.23 (0.76–1.99)	0.83 (0.58–1.18)	0.61 (0.43–0.87)	1.26 (0.83–1.93)	1.20 (0.79–1.83)	1.56 (1.07–2.28)	1.00 (0.42–2.38)	0.76 (0.50–1.16)	0.60 (0.26–1.38)	0.87 (0.62–1.22)	2.85 (1.18–6.87)	0.85 (0.57–1.25)	1.13 (0.77–1.66)

Age and Social support (higher values mean higher social support) are continuous variables.

Comparison category for categorical variables Sex: Female, Region: Berlin, Education: below A-levels, Family status: in relationship, alcohol consumption less than moderate, non-smoker, attention to balanced diet less than moderate, intake of fresh vegetables and fruits on less than 5 days a week, attention physical activity less than moderate, Education of parents: A-levels, attention to balanced diet in childhood less than moderate, attention to physical activity in childhood less than moderate, Parents in favour of pedagogic method: yes, spiritual or religious beliefs of parents: yes.

In general, apart from sociodemographic variables, the actual lifestyle features such as attention towards physical activity showed a stronger impact on outcomes than lifestyle features during childhood like parents' attention towards physical activity.

**Figure 3:**
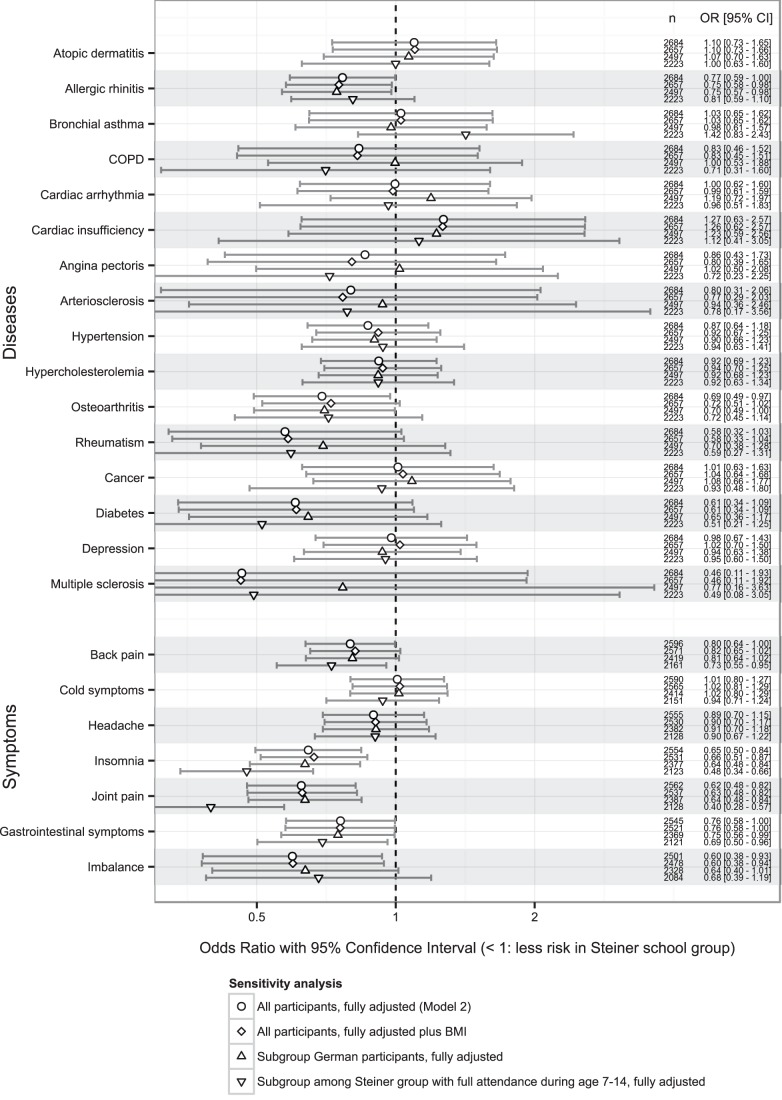
Comparison of effect estimates (odds ratios) in sensitivity analyses.

Comparisons of adjusted odds ratios from sensitivity analyses are given in Figure 3. BMI differences between Steiner group and controls seemed to have no impact on risk of disease report. Restriction of analysis to German citizens attenuates the effect of Steiner school attendance, e.g. for rheumatism and arteriosclerosis. Restriction to the analysis of persons, who reported to have been attending a Steiner school between 7 and 14 years of age, increased the effect considerably in joint pain and insomnia; however, from the 499 Steiner school students excluded in this analysis 333 did not give this information, leaving it unclear whether those attended Steiner schools during the age of 7 to 14 years or not.

The results from the post-hoc sensitivity analyses with imputed datasets show only small differences and are presented in Tables S1 and S2.

## Discussion

We found that the responders from the Steiner school group had lower life time prevalence estimates for some self-reported diseases diagnosed by a physician and documented less self-reported complaints and symptoms as compared to a general population group. Furthermore, former Steiner school attendees had a significantly lower BMI and spent less days in the hospital within the past 12 months. After adjusting for possible confounders, Steiner schools attendees still had significantly lower risk for allergic rhinitis and osteoarthritis as well as symptom burden from back pain, insomnia, joint pain, gastrointestinal symptoms and imbalance. Adjusted analysis showed that beneficial health effects of attendance to Steiner schools are associated with sociodemographic factors as well as with actual and childhood lifestyle.

### Strengths and Limitations

To our knowledge this is the first large comparative survey looking into health status of former Steiner school attendees later in life. Major advantages of the study are the large sample drawn from Steiner schools as well as the random sample from the German population from different German regions, the assessment of different diseases relevant from a public-health perspective, as well as the adjustment for a large number of potentially relevant confounders.

Limitations affect the validity of data collection, as all information was self-reported. Self-reports might be less valid for some diseases than for others. Also, given the retrospective data collection, inaccurate recall might occur, especially in questions regarding childhood. Thus, most missing responses were observed for variables with focus on childhood. However, since absolute numbers of missing values are rather small the impact on effect estimates should be minor.

Furthermore, Steiner attendees may have answered differently than participants in the control group after reading about the research aims which focused on health issues related to their own upbringing lifestyle, although we tried to avoid to draw attention towards this issue, for example by asking more generally about spiritual or religious orientation of participants' parents. In general, given the study design, all results are at risk to be influenced by confounding from unmeasured variables.

In general, the associations between possible risk or protective factors and health outcomes need to be considered as exploratory assessments which are at risk to be substantially biased and can give only little insight in the possible health promoting aspects of Steiner school education. We did not adjust for multiple testing. Thus this must be considered when interpreting p-values. Also, our results are presented as odds ratios and would overestimate the effect if they were interpreted as risk ratios. Considering these points, the presented odds ratios from sociodemographic and lifestyle factors can only serve for hypothesis generation and should not interpreted as exact causal effect estimates.

We fitted the same statistical model on all outcomes to allow comparison of the effect of attending a Steiner school over all outcomes. While for some outcomes the model might include many covariates compared to observed events, for other outcomes this was unproblematic. However, within extensive sensitivity analysis with different modelling strategies (dummy coding instead of dichotomization variables, adjustment for propensity scores, not presented) effect estimates and standard errors of Steiner school attendance did not vary relevantly.

The major challenge in this study was to disentangle the relations between attendance to Steiner school in childhood and possible confounders. Actual socioeconomic status and health behaviour could be a result of Steiner school education or as well be correlated to attending Steiner schools, e.g. with paternal educational status as common cause. Therefore, many differences between both groups can be considered to be possible confounders, but could represent effects of Steiner school education as well. Thus, adjustment for actual lifestyle in statistical analysis could lead to underestimation of effects of Steiner school attendance. Furthermore, we fitted the same model for all outcomes, although effects of variables on outcomes might differ.

Also, it is plausible that relations between Steiner school attendance and lifestyle change over time, especially when keeping in mind that education of study responders took place sometime between the late 1930 s and 2000 s, depending on their respective age. We cannot rule out a time period effect but by adjusting for age we tried to control for this effect in our analyses to a certain degree.

### Comparison to other studies

In some prior studies anthroposophic lifestyle was found to be protective against allergy development [7]–[10], in others this was not the case [14]–[16]. We found that allergies were equally frequent in former Steiner school attendees and control group, but adjustment for potential confounders resulted in a significantly reduced risk of allergic rhinitis among former Steiner school attendees. The prevalence estimates of other diseases that are often caused by allergic reactions such as atopic dermatitis and asthma did not differ between the groups.

Büssing et al. [17] found noteworthy smaller prevalence estimates in former Steiner school attendees compared to the general population for hypertension (10.0% versus 18.6%), osteoarthritis (9.0% versus 26.1%) and rheumatism (3.3% vs. 8.3%), but the authors state that these differences might be due to different modes of data collection. Ruling out this cause of error, we found smaller differences for diseases in our study: hypertension (16.1% versus 19.9%), osteoarthritis (10.8% versus 15.8%) and rheumatism (2.9% versus 4.4%). In adjusted analysis, the difference in osteoarthritis prevalence was statistically significant. In Steiner schools there is a focus on a balance between intellectual, emotional and physical activities compared to a more intellectual focussed education in other schools. Steiner predicts in his philosophy that this could have protective effects especially for arthritic diseases [23]. However, to follow this hypothesis would require a different type of research.

In both groups, lifetime prevalence of diabetes (Steiner 2.5%, control 4.8%) was smaller when compared to recently published estimates from the general population in Germany (7.2%) within the German Health Survey [24]. For depression, we found a lifetime prevalence of 9.7% in the control group, whereas this was considerably higher (male 13.2%, female 21.9%) in the German Health Survey [20] and point prevalence of depression was reported earlier to be 8.1% [24]. This could indicate an underestimation of the true prevalence because of a lack of validity from self-reported assessments.

### Conclusion and future research

This has been the largest and most comprehensive study to date examining the association of attending Steiner schools in childhood with a broad range of illnesses in adults. Our results showed that the prevalence of most widespread diseases including cardiovascular outcomes, diabetes, cancer, depression, COPD and asthma did not differ significantly between former Steiner school attendees compared to adults who attended public schools in childhood. However, osteoarthritis and allergic rhinitis were reported less often by Steiner school attendees considering adjustment for possible confounders including sociodemographic variables, actual and childhood lifestyle features. Interestingly, we found stronger associations of Steiner school attendance with current health complaints: different types of pain, insomnia, imbalance and gastrointestinal symptoms were reported less often after adjustment for possible confounders than in participants who attended other schools. To our knowledge this is the first time that a link between a specific pedagogic framework in childhood and health status in later life was investigated. However, our results must be interpreted with caution since the present analysis was exploratory and bias and residual confounding cannot be ruled out.

Future studies should evaluate possible long-term health benefits of Steiner schools and anthroposophic lifestyle in prospective cohort studies, ideally starting in early childhood with sufficient follow-up into adulthood. Health status and outcomes should be assessed in regular intervals and by objective measurements where possible. To assess the effects of Steiner schools on health and health perception, special emphasis should be put on comparability of groups in terms of socioeconomic status and education. One strategy could be to select controls that had attended some other specialized school and could be more similar to Steiner school attendees. However, parent-reported data is crucial, particularly in young children, to assess lifestyle factors including diet and physical activity outside of school as well as indoor and outdoor environmental exposures.

## Supporting Information

Table S1 **Sensitivity Analysis using multiple imputation.** Combined odds ratios from 100 completely imputed datasets (each n = 2882) of Steiner school attendance, sociodemographic variables, current and childhood lifestyle factors on diseases; from multivariable logistic regression including all factors listed (fully adjusted model 3).
(DOCX)

Table S2 **Sensitivity Analysis using multiple imputation.** Combined odds ratios from 100 completely imputed datasets (each n = 2882) of Steiner school attendance, sociodemographic variables, current and childhood lifestyle factors on diseases; from multivariable logistic regression including all factors listed (fully adjusted model 3).
(DOCX)
