# Supplementary material for: The Effect of Attending Steiner Schools during Childhood on Health in Adulthood: A Multicentre Cross-Sectional Study
Source: PLoS One. 2013 Sep 12;8(9):e73135. doi: 10.1371/journal.pone.0073135 (PMC3771992; doi:10.1371/journal.pone.0073135)
Supplement: Table S1 — Sensitivity Analysis using multiple imputation. Combined odds ratios from 100 completely imputed datasets (each n = 2882) of Steiner school attendance, sociodemographic variables, current and childhood lifestyle factors on diseases; from multivariable logistic regression including all factors listed (fully adjusted model 3). (DOCX) [file pone.0073135.s001.docx]

Table S1: Sensitivity Analysis using multiple imputation. Combined odds ratios from 100 completely imputed datasets (each n = 2882) of Steiner school attendance, sociodemographic variables, current and childhood lifestyle factors on diseases; from multivariable logistic regression including all factors listed (fully adjusted model 3)

|  |  | Sociodemographics | | | | | | | Current lifestyle variables | | | | | | | Childhood lifestyle variables | | | | | | | | |
| --- | --- | --- | --- | --- | --- | --- | --- | --- | --- | --- | --- | --- | --- | --- | --- | --- | --- | --- | --- | --- | --- | --- | --- | --- |
|  | Steiner School | Age (per year) | Sex: Male | Region: Hanover | Region: Nuremberg | Region: Stuttgart | Education: A-level | Family Status: Single | Social Support | Alcohol consumption: moderate and more | Smoker | Attention balanced diet: moderate and more | Fresh vegetables and fruits: 5-7 days per week | Attention physical activity: moderate and more | Physical activities: 5-7 days per week | No siblings | Education Parents: below A-Level | Education Parents: Others/unknown | Parents favour pedagogic method: no | Parents favour pedagogic method: unknown | Parents spiritual or religious beliefs no | Parents spiritual or religious beliefs: unknown | Focus on balanced diet in childhood: moderate and more | Focus on physical activity in childhood: moderate and more |
| Atopic dermatitis | 1.03 (0.69-1.54) | 0.98 (0.97-0.99) | 0.57 (0.41-0.79) | 0.84 (0.55-1.29) | 0.88 (0.58-1.32) | 0.99 (0.66-1.49) | 1.00 (0.70-1.43) | 1.05 (0.76-1.44) | 0.99 (0.92-1.05) | 0.85 (0.62-1.17) | 1.08 (0.77-1.51) | 1.53 (0.93-2.53) | 0.97 (0.70-1.36) | 1.00 (0.73-1.37) | 0.94 (0.63-1.39) | 1.08 (0.72-1.62) | 0.76 (0.53-1.08) | 0.71 (0.29-1.74) | 1.09 (0.72-1.64) | 0.90 (0.40-2.00) | 0.90 (0.66-1.23) | 1.25 (0.48-3.27) | 0.80 (0.54-1.18) | 1.18 (0.81-1.73) |
| Allergic rhinitis | 0.80 (0.62-1.03) | 0.98 (0.98-0.99) | 1.24 (1.02-1.52) | 1.09 (0.82-1.44) | 1.14 (0.87-1.51) | 1.28 (0.98-1.68) | 1.25 (0.99-1.58) | 0.87 (0.70-1.07) | 1.05 (1.00-1.09) | 0.82 (0.67-1.01) | 0.69 (0.55-0.87) | 1.47 (1.08-2.01) | 1.07 (0.86-1.32) | 0.87 (0.71-1.06) | 0.97 (0.76-1.26) | 1.29 (1.00-1.67) | 1.04 (0.83-1.30) | 0.99 (0.57-1.72) | 0.84 (0.65-1.09) | 0.67 (0.40-1.13) | 0.89 (0.73-1.08) | 0.71 (0.33-1.54) | 0.74 (0.58-0.95) | 1.04 (0.82-1.32) |
| Bronchial asthma | 0.88 (0.57-1.36) | 1.00 (0.99-1.01) | 1.24 (0.89-1.75) | 0.93 (0.59-1.47) | 0.84 (0.53-1.35) | 0.82 (0.52-1.29) | 1.09 (0.74-1.61) | 0.83 (0.57-1.20) | 0.99 (0.92-1.06) | 1.00 (0.70-1.43) | 0.89 (0.60-1.32) | 1.17 (0.70-1.98) | 1.08 (0.75-1.56) | 0.96 (0.68-1.36) | 0.94 (0.61-1.47) | 0.97 (0.61-1.53) | 1.10 (0.75-1.61) | 1.37 (0.61-3.08) | 0.90 (0.58-1.39) | 0.83 (0.37-1.87) | 1.08 (0.77-1.52) | 1.05 (0.32-3.46) | 1.13 (0.74-1.74) | 0.89 (0.60-1.32) |
| COPD | 0.84 (0.47-1.49) | 1.04 (1.02-1.05) | 0.69 (0.44-1.09) | 1.12 (0.64-1.96) | 0.52 (0.26-1.04) | 0.85 (0.47-1.55) | 0.97 (0.60-1.56) | 1.06 (0.68-1.65) | 0.93 (0.86-1.00) | 1.21 (0.75-1.96) | 1.75 (1.11-2.76) | 0.78 (0.44-1.37) | 0.62 (0.39-0.97) | 0.67 (0.42-1.06) | 1.11 (0.63-1.96) | 1.04 (0.59-1.83) | 1.13 (0.69-1.86) | 1.41 (0.60-3.31) | 1.14 (0.65-2.00) | 1.53 (0.68-3.46) | 0.99 (0.64-1.54) | 0.98 (0.22-4.35) | 1.07 (0.65-1.78) | 0.90 (0.56-1.43) |
| Cardiac arrhythmia | 0.89 (0.57-1.39) | 1.05 (1.04-1.07) | 1.27 (0.89-1.83) | 0.75 (0.45-1.25) | 0.87 (0.52-1.48) | 1.01 (0.62-1.65) | 1.06 (0.72-1.58) | 1.30 (0.91-1.87) | 0.89 (0.84-0.96) | 1.25 (0.84-1.85) | 0.72 (0.46-1.13) | 1.16 (0.69-1.95) | 0.91 (0.62-1.33) | 0.79 (0.55-1.14) | 1.03 (0.64-1.64) | 1.36 (0.88-2.09) | 1.19 (0.80-1.77) | 1.03 (0.44-2.38) | 0.77 (0.49-1.19) | 0.73 (0.34-1.58) | 0.89 (0.62-1.27) | 0.59 (0.14-2.56) | 0.83 (0.55-1.24) | 1.09 (0.73-1.65) |
| Cardiac insufficiency | 1.23 (0.64-2.37) | 1.06 (1.04-1.08) | 1.22 (0.70-2.11) | 0.50 (0.26-0.98) | 0.57 (0.28-1.15) | 0.26 (0.11-0.60) | 0.69 (0.38-1.25) | 1.40 (0.82-2.40) | 0.96 (0.87-1.06) | 1.55 (0.81-2.94) | 0.44 (0.19-1.01) | 0.85 (0.41-1.77) | 0.75 (0.43-1.31) | 0.70 (0.41-1.21) | 0.60 (0.25-1.44) | 1.09 (0.55-2.17) | 0.86 (0.46-1.59) | 1.31 (0.44-3.89) | 0.96 (0.50-1.87) | 0.66 (0.20-2.14) | 0.95 (0.55-1.62) | 0.00 (0.00-Inf) | 0.88 (0.47-1.65) | 1.35 (0.72-2.52) |
| Angina pectoris | 0.78 (0.42-1.48) | 1.09 (1.06-1.11) | 2.30 (1.38-3.82) | 0.85 (0.43-1.67) | 0.81 (0.39-1.69) | 0.55 (0.26-1.17) | 0.61 (0.35-1.09) | 1.14 (0.68-1.91) | 0.98 (0.89-1.07) | 1.03 (0.60-1.77) | 1.10 (0.60-2.01) | 0.84 (0.42-1.68) | 1.17 (0.69-1.98) | 0.53 (0.32-0.89) | 1.57 (0.85-2.92) | 1.02 (0.53-1.94) | 1.45 (0.80-2.62) | 1.60 (0.59-4.35) | 1.05 (0.56-1.95) | 1.33 (0.55-3.23) | 0.85 (0.52-1.40) | 0.55 (0.07-4.52) | 1.17 (0.64-2.12) | 1.04 (0.59-1.84) |
| Arteriosclerosis | 0.71 (0.30-1.67) | 1.11 (1.08-1.14) | 1.95 (1.01-3.77) | 1.26 (0.43-3.73) | 3.21 (1.11-9.32) | 1.05 (0.33-3.37) | 0.84 (0.38-1.85) | 0.70 (0.34-1.44) | 0.96 (0.85-1.07) | 3.73 (1.49-9.33) | 1.42 (0.63-3.19) | 1.14 (0.45-2.89) | 0.81 (0.42-1.58) | 0.41 (0.20-0.81) | 1.41 (0.59-3.38) | 0.84 (0.33-2.11) | 0.99 (0.44-2.22) | 2.39 (0.73-7.84) | 1.42 (0.63-3.20) | 0.90 (0.27-3.04) | 0.87 (0.45-1.66) | 0.00 (0.00-Inf) | 0.94 (0.43-2.07) | 0.87 (0.42-1.81) |
| Hypertension | 0.86 (0.65-1.15) | 1.07 (1.07-1.08) | 1.40 (1.12-1.76) | 1.15 (0.84-1.58) | 0.94 (0.67-1.32) | 0.90 (0.65-1.24) | 0.91 (0.71-1.17) | 1.12 (0.88-1.42) | 0.97 (0.93-1.01) | 0.89 (0.70-1.13) | 0.93 (0.71-1.21) | 0.77 (0.56-1.07) | 1.01 (0.79-1.30) | 0.76 (0.60-0.95) | 1.02 (0.76-1.36) | 1.04 (0.77-1.40) | 1.21 (0.94-1.55) | 0.80 (0.47-1.39) | 1.01 (0.76-1.34) | 0.78 (0.47-1.31) | 1.04 (0.83-1.31) | 1.15 (0.52-2.52) | 1.07 (0.82-1.39) | 0.84 (0.65-1.08) |
| Hypercholesterolemia | 0.93 (0.71-1.22) | 1.06 (1.05-1.07) | 1.23 (0.99-1.53) | 0.92 (0.67-1.25) | 1.10 (0.80-1.51) | 0.99 (0.73-1.34) | 0.88 (0.69-1.12) | 1.18 (0.94-1.48) | 0.98 (0.94-1.02) | 1.10 (0.87-1.39) | 0.97 (0.75-1.24) | 0.96 (0.71-1.32) | 1.00 (0.79-1.27) | 0.70 (0.56-0.87) | 0.93 (0.70-1.23) | 0.93 (0.69-1.24) | 1.01 (0.79-1.28) | 0.86 (0.51-1.44) | 0.93 (0.71-1.23) | 0.91 (0.56-1.48) | 0.81 (0.65-1.01) | 0.62 (0.27-1.43) | 0.87 (0.67-1.12) | 1.00 (0.78-1.28) |
| Osteoarthritis | 0.71 (0.52-0.98) | 1.08 (1.07-1.09) | 0.70 (0.54-0.91) | 0.98 (0.66-1.43) | 1.49 (1.01-2.21) | 1.53 (1.06-2.23) | 0.90 (0.68-1.19) | 1.04 (0.79-1.35) | 0.96 (0.91-1.01) | 1.02 (0.78-1.34) | 1.01 (0.75-1.37) | 0.85 (0.59-1.22) | 1.15 (0.87-1.53) | 0.90 (0.70-1.17) | 1.29 (0.94-1.77) | 0.94 (0.67-1.32) | 1.14 (0.86-1.50) | 1.13 (0.64-2.00) | 1.10 (0.80-1.51) | 0.91 (0.53-1.56) | 1.08 (0.84-1.40) | 1.12 (0.46-2.70) | 0.88 (0.65-1.19) | 1.50 (1.11-2.02) |
| Rheumatism | 0.59 (0.34-1.02) | 1.06 (1.04-1.07) | 0.81 (0.52-1.26) | 1.09 (0.59-2.03) | 0.76 (0.38-1.54) | 1.65 (0.91-2.98) | 0.90 (0.56-1.45) | 1.63 (1.08-2.47) | 0.94 (0.87-1.02) | 1.18 (0.73-1.91) | 1.35 (0.84-2.17) | 0.64 (0.36-1.13) | 1.01 (0.63-1.61) | 0.57 (0.36-0.88) | 1.67 (1.01-2.76) | 0.73 (0.39-1.38) | 0.87 (0.54-1.41) | 1.60 (0.73-3.53) | 0.75 (0.44-1.27) | 0.81 (0.35-1.87) | 0.81 (0.53-1.25) | 1.23 (0.35-4.38) | 1.14 (0.68-1.90) | 0.91 (0.57-1.47) |
| Cancer | 0.90 (0.57-1.40) | 1.07 (1.05-1.08) | 0.90 (0.62-1.30) | 1.51 (0.90-2.51) | 0.89 (0.50-1.60) | 1.06 (0.62-1.81) | 0.79 (0.53-1.17) | 1.06 (0.73-1.52) | 1.03 (0.96-1.10) | 1.51 (1.01-2.27) | 1.17 (0.76-1.79) | 0.84 (0.50-1.42) | 0.83 (0.57-1.22) | 1.13 (0.79-1.61) | 0.79 (0.48-1.28) | 1.01 (0.62-1.62) | 0.98 (0.66-1.47) | 1.92 (0.95-3.91) | 1.00 (0.64-1.54) | 0.35 (0.14-0.89) | 0.96 (0.67-1.37) | 0.75 (0.17-3.31) | 1.16 (0.76-1.78) | 0.97 (0.65-1.46) |
| Diabetes | 0.54 (0.31-0.94) | 1.07 (1.06-1.09) | 1.91 (1.24-2.96) | 1.10 (0.60-2.03) | 0.84 (0.42-1.65) | 1.17 (0.62-2.17) | 0.64 (0.40-1.02) | 1.08 (0.70-1.68) | 1.03 (0.95-1.11) | 1.21 (0.77-1.90) | 1.33 (0.82-2.15) | 0.70 (0.40-1.23) | 0.68 (0.43-1.05) | 0.77 (0.50-1.18) | 1.20 (0.69-2.08) | 1.43 (0.86-2.38) | 0.93 (0.58-1.49) | 0.83 (0.33-2.10) | 0.85 (0.51-1.43) | 1.01 (0.45-2.24) | 1.23 (0.80-1.88) | 2.30 (0.72-7.31) | 1.08 (0.65-1.79) | 1.09 (0.67-1.78) |
| Depression | 0.97 (0.68-1.39) | 1.01 (1.00-1.02) | 0.46 (0.34-0.62) | 0.67 (0.45-1.00) | 0.93 (0.65-1.35) | 0.92 (0.64-1.32) | 1.27 (0.93-1.74) | 1.38 (1.05-1.81) | 0.84 (0.80-0.89) | 0.88 (0.66-1.18) | 1.94 (1.45-2.58) | 1.21 (0.82-1.79) | 0.73 (0.55-0.98) | 0.83 (0.62-1.10) | 1.24 (0.88-1.76) | 1.20 (0.84-1.71) | 1.03 (0.75-1.40) | 1.45 (0.79-2.66) | 0.98 (0.68-1.40) | 0.65 (0.32-1.28) | 0.73 (0.55-0.97) | 0.73 (0.28-1.93) | 0.80 (0.58-1.12) | 0.82 (0.61-1.12) |
| Multiple sclerosis | 0.42 (0.10-1.71) | 1.00 (0.97-1.03) | 0.58 (0.21-1.63) | 0.39 (0.07-2.10) | 0.77 (0.19-3.03) | 2.07 (0.66-6.54) | 0.66 (0.23-1.94) | 1.07 (0.41-2.79) | 0.93 (0.78-1.12) | 1.80 (0.56-5.82) | 1.70 (0.65-4.49) | 0.71 (0.21-2.39) | 0.67 (0.24-1.86) | 0.34 (0.11-1.08) | 0.81 (0.18-3.67) | 0.54 (0.12-2.42) | 0.84 (0.29-2.42) | 0.46 (0.05-4.46) | 0.95 (0.28-3.17) | 2.10 (0.42-10.51) | 1.28 (0.50-3.27) | 0.00 (0.00-Inf) | 1.45 (0.42-5.00) | 1.38 (0.44-4.35) |
