# Supplementary material for: The Effect of Attending Steiner Schools during Childhood on Health in Adulthood: A Multicentre Cross-Sectional Study
Source: PLoS One. 2013 Sep 12;8(9):e73135. doi: 10.1371/journal.pone.0073135 (PMC3771992; doi:10.1371/journal.pone.0073135)
Supplement: Table S2 — Sensitivity Analysis using multiple imputation. Combined odds ratios from 100 completely imputed datasets (each n = 2882) of Steiner school attendance, sociodemographic variables, current and childhood lifestyle factors on diseases; from multivariable logistic regression including all factors listed (fully adjusted model 3). (DOCX) [file pone.0073135.s002.docx]

Table S2: Sensitivity Analysis using multiple imputation: Combined odds ratios from 100 completely imputed datasets (each n = 2882) of Steiner school attendance, sociodemographic variables, current and childhood lifestyle factors on diseases; from multivariable logistic regression including all factors listed (fully adjusted model 3)

|  | |  | Sociodemographics | | | | | | | Actual lifestyle variables | | | | | | | Childhood lifestyle variables | | | | | | | | |
| --- | --- | --- | --- | --- | --- | --- | --- | --- | --- | --- | --- | --- | --- | --- | --- | --- | --- | --- | --- | --- | --- | --- | --- | --- | --- |
|  | | Steiner School | Age (per year) | Sex: Male | Region: Hanover | Region: Nuremberg | Region: Stuttgart | Education: A-level | Family Status: Single | Social Support | Alcohol consummation: moderate and more | Smoker | Attention balanced diet: moderate and more | Fresh vegetables and fruits: 5-7 days per week | Attention physical activity: moderate and more | Physical activities: 5-7 days per week | No siblings | Education Parents: below A-Level | Education Parents: Others/unknown | Parents favour pedagogic method: no | Parents favour pedagogic method: unknown | Parents spiritual or religious beliefs no | Parents spiritual or religious beliefs: unknown | Focus on balanced diet in childhood: moderate and more | Focus on physical activity in childhood: moderate and more |
| Back pain | | 0.76 (0.61-0.94) | 1.01 (1.01-1.02) | 0.63 (0.53-0.75) | 1.11 (0.88-1.40) | 1.27 (1.01-1.60) | 1.03 (0.82-1.29) | 0.78 (0.65-0.93) | 0.97 (0.81-1.15) | 0.94 (0.91-0.97) | 0.96 (0.81-1.14) | 1.41 (1.17-1.69) | 0.97 (0.76-1.23) | 0.86 (0.72-1.02) | 0.71 (0.60-0.85) | 1.06 (0.86-1.32) | 0.99 (0.79-1.24) | 1.12 (0.93-1.35) | 1.13 (0.74-1.71) | 0.97 (0.78-1.20) | 1.23 (0.83-1.82) | 1.03 (0.87-1.22) | 1.21 (0.68-2.13) | 0.90 (0.73-1.11) | 0.99 (0.81-1.20) |
| Cold symptoms | | 1.00 (0.80-1.25) | 0.99 (0.98-0.99) | 0.87 (0.73-1.04) | 0.94 (0.74-1.19) | 0.87 (0.68-1.10) | 0.76 (0.60-0.96) | 1.24 (1.02-1.52) | 1.09 (0.91-1.31) | 0.95 (0.92-0.99) | 0.92 (0.76-1.10) | 1.30 (1.07-1.57) | 1.15 (0.89-1.48) | 1.07 (0.88-1.29) | 0.89 (0.75-1.07) | 0.86 (0.69-1.08) | 1.02 (0.81-1.29) | 1.12 (0.92-1.36) | 2.16 (1.40-3.32) | 0.96 (0.76-1.21) | 1.25 (0.83-1.88) | 0.96 (0.80-1.14) | 1.38 (0.76-2.49) | 0.87 (0.70-1.08) | 1.05 (0.85-1.29) |
| Headache | | 0.83 (0.65-1.06) | 0.98 (0.97-0.98) | 0.41 (0.34-0.50) | 1.16 (0.90-1.49) | 0.89 (0.69-1.14) | 0.92 (0.72-1.18) | 0.79 (0.64-0.97) | 0.95 (0.78-1.15) | 0.93 (0.90-0.97) | 1.10 (0.90-1.34) | 1.12 (0.91-1.37) | 0.97 (0.74-1.26) | 0.76 (0.63-0.93) | 0.73 (0.61-0.89) | 0.93 (0.74-1.19) | 1.19 (0.93-1.51) | 1.13 (0.92-1.39) | 1.21 (0.77-1.91) | 0.89 (0.70-1.13) | 1.02 (0.67-1.56) | 0.96 (0.80-1.16) | 1.33 (0.72-2.46) | 0.91 (0.72-1.14) | 0.94 (0.76-1.17) |
| Insomnia | | 0.63 (0.49-0.81) | 1.02 (1.01-1.02) | 0.63 (0.51-0.77) | 1.10 (0.84-1.44) | 1.02 (0.78-1.34) | 0.94 (0.72-1.23) | 0.85 (0.68-1.05) | 1.23 (1.01-1.50) | 0.90 (0.86-0.93) | 0.98 (0.80-1.21) | 1.19 (0.96-1.48) | 0.94 (0.72-1.25) | 0.87 (0.70-1.07) | 0.73 (0.60-0.90) | 1.18 (0.92-1.52) | 1.13 (0.88-1.46) | 1.15 (0.93-1.43) | 1.43 (0.91-2.25) | 0.73 (0.57-0.94) | 0.83 (0.53-1.29) | 0.93 (0.76-1.13) | 1.08 (0.55-2.12) | 1.12 (0.88-1.42) | 0.93 (0.74-1.17) |
| Joint pain | | 0.57 (0.44-0.74) | 1.05 (1.04-1.05) | 0.89 (0.73-1.09) | 1.08 (0.81-1.43) | 1.25 (0.93-1.66) | 1.05 (0.79-1.39) | 0.66 (0.53-0.82) | 1.06 (0.85-1.31) | 0.96 (0.92-1.00) | 1.12 (0.91-1.39) | 1.30 (1.04-1.63) | 0.96 (0.72-1.27) | 0.87 (0.70-1.08) | 0.72 (0.59-0.89) | 1.20 (0.93-1.55) | 0.91 (0.70-1.19) | 1.01 (0.81-1.26) | 1.38 (0.88-2.15) | 0.92 (0.71-1.18) | 1.05 (0.67-1.63) | 1.09 (0.89-1.33) | 1.83 (0.95-3.55) | 1.02 (0.81-1.30) | 1.01 (0.80-1.27) |
| Gastrointestinal symptoms | | 0.74 (0.57-0.96) | 1.00 (1.00-1.01) | 0.74 (0.60-0.91) | 1.01 (0.76-1.34) | 1.10 (0.83-1.45) | 0.96 (0.73-1.27) | 0.91 (0.72-1.14) | 1.16 (0.94-1.43) | 0.92 (0.89-0.96) | 1.08 (0.87-1.34) | 1.17 (0.93-1.46) | 1.09 (0.82-1.46) | 0.84 (0.67-1.04) | 0.68 (0.55-0.84) | 0.85 (0.65-1.12) | 0.87 (0.66-1.15) | 0.87 (0.69-1.09) | 1.20 (0.75-1.91) | 0.83 (0.64-1.07) | 0.92 (0.58-1.47) | 0.95 (0.78-1.17) | 1.03 (0.51-2.08) | 0.92 (0.72-1.18) | 1.05 (0.82-1.32) |
| Imbalance | | 0.50 (0.32-0.77) | 1.03 (1.02-1.04) | 0.74 (0.53-1.03) | 1.02 (0.66-1.58) | 0.97 (0.61-1.52) | 0.68 (0.42-1.08) | 0.95 (0.67-1.36) | 1.38 (0.99-1.91) | 0.93 (0.88-0.99) | 1.54 (1.06-2.24) | 1.02 (0.70-1.47) | 1.30 (0.82-2.08) | 0.77 (0.55-1.08) | 0.60 (0.42-0.83) | 1.33 (0.88-2.00) | 1.14 (0.76-1.71) | 1.51 (1.05-2.18) | 0.95 (0.42-2.17) | 0.70 (0.47-1.04) | 0.53 (0.25-1.14) | 0.90 (0.65-1.25) | 2.96 (1.28-6.84) | 0.83 (0.57-1.22) | 1.09 (0.75-1.57) |
|  | Age and Social support (higher values mean higher social support) are continuous variables.  Comparison category for categorical variables Sex: Female, Region: Berlin, Education: below A-levels, Family status: in relationship, alcohol consumption less than moderate, non-smoker, attention to balanced diet less than moderate, intake of fresh vegetables and fruits on less than 5 days a week, attention physical activity less than moderate, Education of parents: A-levels, attention to balanced diet in childhood less than moderate, attention to physical activity in childhood less than moderate, Parents in favour of pedagogic method: yes, spiritual or religious beliefs of parents: yes | | | | | | | | | | | | | | | | | | | | | | | | |
